# Complete Genome Sequence of the Novel Duck Circovirus Strain GH01 from Southwestern China

**DOI:** 10.1128/genomeA.00166-12

**Published:** 2013-02-07

**Authors:** Zhilong Zhang, Renyong Jia, Mingshu Wang, Yanyan Lu, Dekang Zhu, Shun Chen, Zhongqiong Yin, Yin Wang, Xiaoyue Chen, Anchun Cheng

**Affiliations:** a Institute of Preventive Veterinary Medicine, Sichuan Agricultural University, Chengdu City, Sichuan, China; b Avian Disease Research Center, Sichuan Agricultural University, Ya’an, Sichuan, China; c Key Laboratory of Animal Disease and Human Health of Sichuan Province, Chengdu City, Sichuan, China

## Abstract

A novel duck circovirus (DuCV) strain, designated GH01, was isolated from ducklings in southwestern China. We report the genome sequence of GH01 and the genomic organization and genetic relationship to other DuCVs. The availability of the genome sequence will be helpful to investigations of epidemiology and the evolutionary biology of this organism and the development of preventive vaccines.

## GENOME ANNOUNCEMENT

Duck circovirus (DuCV) is a small nonenveloped virus with a monomeric single-strand circular DNA genome. The virus was initially discovered in Germany in 2003 (1) and was subsequently identified in the United States, Taiwan, China, and other areas (2–4). Nowadays, DuCV is highly prevalent in the domestic duck population and is associated with considerable economic losses, with the main clinical symptoms including immunosuppression and stunting and feather abnormalities. DuCV has been tentatively clustered in the *Circoviridae* family genus *Circovirus*, according to the eighth report of the International Committee on Taxonomy of Viruses (ICTV) (5). The deficiency of genome sequence and genomic organization information has limited the progress of DuCV taxonomy.

Here, we report the complete genome sequence of the DuCV strain GH01, which was isolated from infected ducks in Sichuan Province, southwestern China; the main clinical symptoms were stunting and feather abnormalities in ducklings. This study was approved by the National Institute of Animal Health Animal Care and Use Committee at Sichuan Agricultural University (approval number 2012-032). The nucleotide sequence of GH01 was amplified through PCR, and the PCR products were purified and cloned into the pMD18-T vector (TaKaRa) and then sequenced by using classical dideoxy Sanger sequencing (TaKaRa, Dalian, China) and assembled by Lasergene 7.1 software packages.

The complete genome of GH01 comprises 1,988 nucleotides (nt) as monomeric single-strand circular DNA with a G+C content of 48.4%. Six open reading frames (ORFs) were identified according to the major criteria, a minimum length of 60 bp, an ATG start codon, and less than 60% overlap with adjacent ORFs, with the use of Lasergene 7.1 software packages. Based on similarities and the presence of conserved domains, two major ORFs, V1 (*rep*) and C1 (*cap*), on complementary strands in opposite orientations were predicted to encode the viral replicase (292 amino acids) and capsid protein (257 amino acids). Similar to related circoviruses, GH01 has two intergenic noncoding regions that are 111- and 224-nt long. The shorter noncoding region between the two major ORFs contains a thermodynamically stable stem-loop for initiation of rolling-circle replication, with a characteristic nonamer motif (TATTATTAC) and two hexamer motifs (ACTCCG) that serve as binding sites for the replicases.

A phylogenetic tree based on the entire genome sequence of duck circovirus indicates that GH01 is more closely related to FJCL311 (GenBank accession number GQ423743.1), an isolate from southern China, than the strains isolated from other areas of China, Germany, and the United States. This report will help further our understanding of the molecular characteristics of duck circovirus and the epidemiology of infection in southwestern China.

So far, no effective preventive vaccination against DuCV in poultry has been used, and the nature of circulating DuCV has largely remained elusive, with outbreaks of infection continuously occurring in China. Our hope in submitting this report is that these data will facilitate investigations by others in the virology community into the epidemiology, evolutionary biology, and pathobiology of circovirus infection. Moreover, the availability of the DuCV genome sequence potentially facilitates the development of new and more effective vaccines for DuCV.

### Nucleotide sequence accession number.

The complete genome sequence of DuCV strain GH01 is available in GenBank under the accession number JX499186.
